# *In vivo* impact of presynaptic calcium channel dysfunction on motor axons in episodic ataxia type 2

**DOI:** 10.1093/brain/awv380

**Published:** 2016-01-27

**Authors:** Susan E. Tomlinson, S. Veronica Tan, David Burke, Robyn W. Labrum, Andrea Haworth, Vaneesha S. Gibbons, Mary G. Sweeney, Robert C. Griggs, Dimitri M. Kullmann, Hugh Bostock, Michael G. Hanna

**Affiliations:** ^1^ 1 Sydney Medical School, University of Sydney, Australia; ^2^ 2 Department of Neurology, St Vincent’s Hospital, Sydney, Australia; ^3^ 3 Institute of Neurology, University College London and MRC Centre for Neuromuscular Disease, Queen Square, UK; ^4^ 4 Department of Neurology, Royal Prince Alfred Hospital, Sydney, Australia; ^5^ 5 Neurogenetics Unit, National Hospital for Neurology, Queen Square, UK; ^6^ 6 University of Rochester, New York, USA

**Keywords:** channelopathy, axonal excitability

## Abstract

Ion channel dysfunction causes a range of neurological disorders by altering transmembrane ion fluxes, neuronal or muscle excitability, and neurotransmitter release. Genetic neuronal channelopathies affecting peripheral axons provide a unique opportunity to examine the impact of dysfunction of a single channel subtype in detail *in vivo.* Episodic ataxia type 2 is caused by mutations in *CACNA1A*, which encodes the pore-forming subunit of the neuronal voltage-gated calcium channel Ca_v_2.1. In peripheral motor axons, this channel is highly expressed at the presynaptic neuromuscular junction where it contributes to action potential-evoked neurotransmitter release, but it is not expressed mid-axon or thought to contribute to action potential generation. Eight patients from five families with genetically confirmed episodic ataxia type 2 underwent neurophysiological assessment to determine whether axonal excitability was normal and, if not, whether changes could be explained by Ca_v_2.1 dysfunction. New mutations in the *CACNA1A* gene were identified in two families. Nerve conduction studies were normal, but increased jitter in single-fibre EMG studies indicated unstable neuromuscular transmission in two patients. Excitability properties of median motor axons were compared with those in 30 age-matched healthy control subjects. All patients had similar excitability abnormalities, including a high electrical threshold and increased responses to hyperpolarizing (*P* < 0.00007) and depolarizing currents (*P* < 0.001) in threshold electrotonus. In the recovery cycle, refractoriness (*P* < 0.0002) and superexcitability (*P* < 0.006) were increased. Ca_v_2.1 dysfunction in episodic ataxia type 2 thus has unexpected effects on axon excitability, which may reflect an indirect effect of abnormal calcium current fluxes during development.

## Teaser

Episodic ataxia type 2 is caused by mutations in CACNA1A, which encodes the pore-forming subunit of the voltage-gated calcium channel Ca_v_2.1. Tomlinson *et al*. reveal altered peripheral axonal excitability in patients compared to controls. The changes may reflect malformation of the nodes of Ranvier, as demonstrated in animal models.

## Graphical abstract

**Figure:**
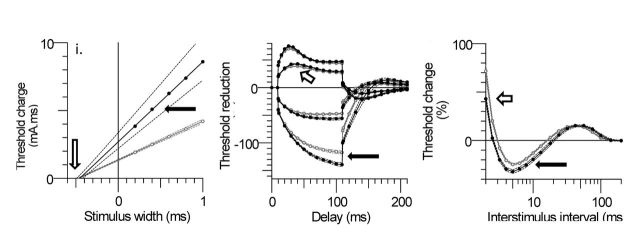
Episodic ataxia type 2 is caused by mutations in CACNA1A, which encodes the pore-forming subunit of the voltage-gated calcium channel Ca_v_2.1. Tomlinson *et al*. reveal altered peripheral axonal excitability in patients compared to controls. The changes may reflect malformation of the nodes of Ranvier, as demonstrated in animal models.

## Introduction

Episodic ataxia type 2 (EA2) is an autosomal dominant disorder caused by mutations in the *CACNA1A* gene, which encodes the α1A subunit of the presynaptic P/Q-type calcium channel Ca_v_2.1 (Gancher and Nutt, 1986; Ophoff *et al.*, 1996). The channel is widely expressed in the CNS, particularly in the cerebellum and hippocampus (Vacher *et al.*, 2008), and in spinal motor neurons (Westenbroek *et al.*, 1988). Large myelinated motor axons in peripheral nerve express Ca_v_2.1 calcium channels at the presynaptic membrane at the neuromuscular junction, where, together with N-type Ca_v_2.2 channels, they trigger action potential-evoked neurotransmitter release (Day *et al.*, 1997), but in rodents, Ca_v_2.1 channels are not normally expressed in peripheral axons between the cell body and the neuromuscular junction (Ousley and Froehner, 1994; Vogel and Schwarz, 1995; Plant *et al.*, 1998). This also seems to be true for human axons (Schwarz *et al.*, 1995; Protti *et al.*, 1996). However, Ca_v_2.1 channels have been reported during development in neonatal rodent optic nerve axons (‘developing central axons’), where they contribute to normal action potential propagation (Alix *et al.*, 2008). As indicated by Alix *et al.* (2008), the α1A subunits that form P/Q channels are transiently clustered in the axolemma of immature axons and are expressed where the underlying vesiculotubular complex is fusing with the axolemma, including regions where development of node of Ranvier is occurring. Mutations of either the α1A subunit or the α2δ2 subunit resulted in malformed nodes of Ranvier in mice, manifesting morphologically as loss of nodal protein localization, disruption of the nodal membrane and build-up of vesicles under the nodal axolemma (Alix *et al.*, 2008). It was hypothesized that disrupting calcium channel localization has either a ‘direct’ effect on node of Ranvier development due to a disruption in vesicle docking at the nodal axolemma, or an ‘indirect’ effect, stemming from a reduction in axonal excitability (Alix *et al.*, 2008).

Patients with EA2 manifest an intermittent cerebellar disturbance, which can last hours to days (Jen *et al.*, 2007). Some patients develop fixed or progressive cerebellar features, and there is an increased incidence of epilepsy in patients with EA2 (Rajakulendran *et al.*, 2010). The condition is allelic with spinocerebellar ataxia type 6 (SCA6), and familial hemiplegic migraine type 1 (FHM1), and there may be clinical overlap between these disorders (Battistini *et al.*, 1999; Jen *et al.*, 1999; Kors *et al.*, 2004). Intermittent myasthaenia-like weakness has been reported in some patients with EA2 (Jen *et al.*, 1999). During nerve conduction studies in these patients, repetitive stimulation and voluntary contractions can produce features suggestive of variable presynaptic endplate function (Jen *et al.*, 2001; Melzer *et al.*, 2010), and there may be neurophysiological evidence of a disturbance of transmission across the neuromuscular junction (Maselli *et al.*, 2003).

We have previously reported highly stereotypic changes in the excitability of motor axons in the median nerve of patients with episodic ataxia type 1 (EA1), which is caused by loss-of-function mutations of the potassium channel gene *KCNA1* (Tomlinson *et al.*, 2010). These changes in excitability are not unexpected, because the K_v_1.1 subunit encoded by *KCNA1* is expressed in the juxtaparanodal membrane of motor axons, and EA1 patients commonly exhibit neuromyotonia. We have also observed abnormal axonal excitability in subjects with mutations in *KCNQ2* channel gene responsible for slower K^+^ currents. These subjects had a history of benign familial neonatal epilepsy, but were asymptomatic when studied and did not have neuromyotonia (Tomlinson *et al.*, 2012).

The purpose of the present study was to evaluate whether EA2 patients would have changes in axonal excitability, even though calcium channels are not normally expressed in mid-axon (at the site of stimulation), and axonal function is normal in routine nerve conduction studies.

## Materials and methods

Eight patients with a clinical phenotype of EA2 were studied in the UK, USA and Australia between 2007 and 2009 by the same clinician (S.T.), who performed the nerve excitability studies on patients and control subjects contemporaneously. All subjects were over the age of 18 and provided written informed consent. Ethical approval was obtained from the Joint National Hospital for Neurology and Neurosurgery/University College London Ethics Committee, The University of Rochester Ethics Committee, and the University of Sydney Human Research Ethics Committee. They were assessed clinically and, where possible, using nerve conduction studies (including repetitive stimulation), EMG, single fibre EMG, and stimulated single fibre EMG, according to a previously described protocol (Mills, 2005; Tomlinson *et al.*, 2009). Medications were not changed for assessment. Two subjects (Subjects 3 and 5) were not taking acetazolamide at the time of clinical or neurophysiological assessment. Genomic DNA was extracted from peripheral blood leucocytes using standard procedures. Probands were screened for *CACNA1A* mutations [NCBI transcript NM_001127221.1 (on LRG_7)] by standard bidirectional Sanger sequencing of all coding exons and exon–intron boundaries (primer sequences available on request). Dosage analysis for *CACNA1A* exonic deletions and duplications was performed by multiplex ligation-dependent probe amplification (MRC).

### Nerve excitability studies

In previous publications, the terms ‘nerve excitability studies’ and ‘axonal excitability studies’ have been used interchangeably, and the latter will be used in this article. Excitability studies were performed on motor axons in the median nerve using the TROND E protocol (Kiernan *et al.*, 2000; Burke *et al.*, 2001), and were analysed with Qtrac software (©UCL Institute of Neurology, UK). The studies were performed at attack-free times, not during an ictus. During a 15-min study, the median nerve was stimulated at the wrist with the anode placed 10 cm more proximally over muscle, lateral to the median nerve. The compound muscle action potential (CMAP) was recorded over the abductor pollicis brevis. The reference electrode was placed distally, over the distal interphalangeal joint of the thumb. The Qtrac program generated stimulus waveforms via a data acquisition board (National Instruments; NI USB 6221), which were delivered to the subject by a DS5 isolated linear bipolar current stimulator (Digitimer). There were four parts to the test. Nerve excitability studies first evaluated the stimulus–response relationship. From the stimulus–response curve, a ‘target’ response was set to the steepest part of the curve, where the thresholds of the motor axons are most concentrated, ∼40% of the maximal CMAP amplitude.

Subsequent measurements in the protocol were based on ‘threshold tracking’, in which the current required to produce the target CMAP is referred to as a ‘threshold’ for that CMAP. Conditioning stimuli were applied to the nerve, and the change in stimulus required to produce the target CMAP response was ‘tracked’. There were three manipulations designed to measure: (i) strength-duration properties; (ii) the responses to subthreshold conditioning stimuli; and (iii) the responses to supramaximal conditioning stimuli. Each provides information about different influences on membrane potential and ion channel function.

#### Strength–duration properties

Strength–duration measurements of nerve membrane reflect properties of the nodes of Ranvier, and can provide insight into persistent Na^+^ conductances active at the resting potential (Bostock and Rothwell, 1997). The measurement was made using unconditioned test stimuli of different duration (0.2, 0.4, 0.6, 0.8 and 1.0 ms). As stimulus duration increased, a smaller current was required to produce the target CMAP. Using these measurements, rheobase and the strength–duration time constant (τsd) were calculated from a plot of stimulus charge against stimulus duration.

#### Threshold electrotonus

During threshold electrotonus (TE)*,* subthreshold polarizing currents were applied for 100 ms, and the change in threshold was tested at different times, before and during the 100 ms polarizing currents and for 100 ms afterwards. The conditioning stimuli were hyperpolarizing (TEh) or depolarizing (TEd), and were set at strengths of 20% (TEd^20^ or TEh^20^) and 40% (TEd^40^ or TEh^40^) of the unconditioned threshold current (which was monitored throughout the recording). Threshold electrotonus can provide insight into the state of membrane polarization and the activity of some voltage-dependent ion channels: nodal Na^+^ channels and slow K^+^ channels, and internodal K^+^ and hyperpolarization-activated, cyclic nucleotide-gated (HCN) channels (Bostock *et al.*, 1998; Howells *et al.*, 2012).

#### Current/threshold relationship

The current/threshold (I/V) relationship measured the change in threshold at the end of a 200 ms conditioning current, which was varied in 10% steps from 50% (depolarizing) to −100% (hyperpolarizing). The resting I/V slope is the slope of the current–threshold relationship at resting membrane potential. The minimum I/V slope is the minimum slope of the current–threshold curve. The hyperpolarizing I/V slope: slope of the current–threshold relationship for hyperpolarizing currents of −80% to −100% of threshold. These data allowed rectifying currents to be assessed; K^+^ currents with depolarization and inwardly rectifying HCN currents (I*_h_*) with hyperpolarization. The slope of the I/V curve is analogous to conductance.

#### Recovery cycle

The recovery cycle consists of the relative refractory period (RRP, largely reflecting recovery of nodal Na^+^ channels from inactivation), the superexcitable period (due to the depolarizing after potential, which can be affected by the activity of fast K^+^ channels), and the late subexcitable period (reflecting the slowly decaying hyperpolarization following slow K^+^ channel activation) (Bostock *et al.*, 1998; Burke *et al.*, 2009; Howells *et al.*, 2012). In the TROND protocol, the recovery cycle was measured using a supramaximal conditioning stimulus. The change in threshold was tracked at intervals up to 200 ms after the conditioning stimulus. The conditioning potential was subtracted on-line from the response to the conditioning and test stimulus pair so that accurate measurements could be made at short conditioning-test intervals.

### Statistical analysis

Control data for nerve excitability studies were obtained from age-matched subjects from a database of healthy volunteers (Table 2). Statistical analysis was performed by the QTRACP software (version 23/10/2012). Differences in mean nerve excitability measures between patients and controls were assessed by Welch’s unequal variance *t*-test. Because we were interested in which measurements were different, rather than in whether any measurements were different, Bonferroni correction was not applied, and *P* < 0.05 was considered significant.

### Mathematical modelling

A mathematical model of axonal excitability was applied to the data generated from the studies (Bostock *et al.*, 1995; Kiernan *et al.*, 2005*a*; Jankelowitz *et al.*, 2007; Howells *et al.*, 2012). The means and standard deviations (SDs) of each recorded data point were evaluated for the 30 normal subjects and the eight patients. The conductances of the model were first adjusted to minimize the discrepancy between the model and the mean control recordings, where discrepancy was defined as the weighted sum of the error terms: ([x_m_ − x_n_] / s_n_)^2^, where x_m_ is the threshold of the model, x_n_ is the mean, and s_n_ is the standard deviation of the thresholds for the real nerves. The weights were the same for all thresholds of the same type (e.g. recovery cycle) and were chosen to give total weights to the four different types of threshold measurement: threshold electrotonus, current–threshold relation, recovery cycle, and strength–duration time constant in the ratio 4:2:2:1. As in previous modelling studies, these ratios reflect, albeit crudely, that the four threshold electrotonus traces provide more information about membrane properties than the current/threshold relationship or the recovery cycle, which in turn provide more information than the strength–duration relationship. The ‘control’ model was achieved when the discrepancy could not be further reduced by changing any of 29 parameters in the model, alone or in combination. Then, for each of the 17 parameters in the model (comprising 10 conductances, three capacitances, 15 channel gating parameters and the resting membrane potential), either alone or in combination. To explore possible explanations for the abnormal excitability properties of axons in EA2 a different strategy was used: selected model parameters were tested one at a time to see how well changes in a single parameter could account for the abnormalities. The modelling and the fitting of the model to the control and patient recordings were carried out by the MEMFIT function within the QTRACP program (version 23/10/2012).

## Results

### Subject characteristics

Eight patients with genetically confirmed EA2 from five families were studied. Clinical features are summarized in Table 1. Subjects 1 and 2 were dizygotic twins. A family history of EA2 was defined in seven patients. All subjects gave a history of intermittent cerebellar dysfunction lasting hours to days, triggered by stress/emotion, intercurrent illness and vigorous exertion or sudden movement. All patients demonstrated a clinical response to acetazolamide but this was discontinued in two because of side effects (Subject 3 due to renal calculi; Subject 5 due to thrombocytopaenia). Subjects 3 and 5 were both from Family 2 and reported feeling muscle weakness in association with their ataxic episodes. 

**Table 1:** Clinical and genetic features of patients with EA2

Family (mutation)	Subject	Age, sex	Family history	Clinical features
**A** c.3992+1G>A p.?	1	39; M	Yes	Onset age 6. Up to 4 episodes/week. Subtle interictal cerebellar findings.
	2	39; F	Yes	Onset age 6. Up to 4 episodes a month. Normal interictal exam.
**B** c.5137–10_–2delAATTCCACA p.?	3	66; M	Yes	Onset age 7. Up to 4 episodes/week. Minor interictal cerebellar findings. Acetazolamide ceased due to renal calculi.
	4	40; M	Yes	Onset age 4. Up to 4 episodes/week. Normal exam.
	5	34; F	Yes	Onset age 8. Up to 2 episodes/week. Normal exam. Acetazolamide ceased due to thrombocytopaenia.
**C** c.3695+1G>A p.?	6	37; F	Yes	Onset age 6. Up to 4 episodes/month. Minor interictal cerebellar findings.
**D** c.5455C>T p.Arg1819*	7	21; M	No	Onset age 5 when started sports. Up to 2 episodes/week. Requires 4-AP in addition to acetazolamide.
**E** c.835C>T p.(Arg279Cys)	8	48; F	Yes	Onset age 5. Normal interictal exam. Requires dichlorphenamide and topirimate in addition to acetazolamide.

**Figure 1:**
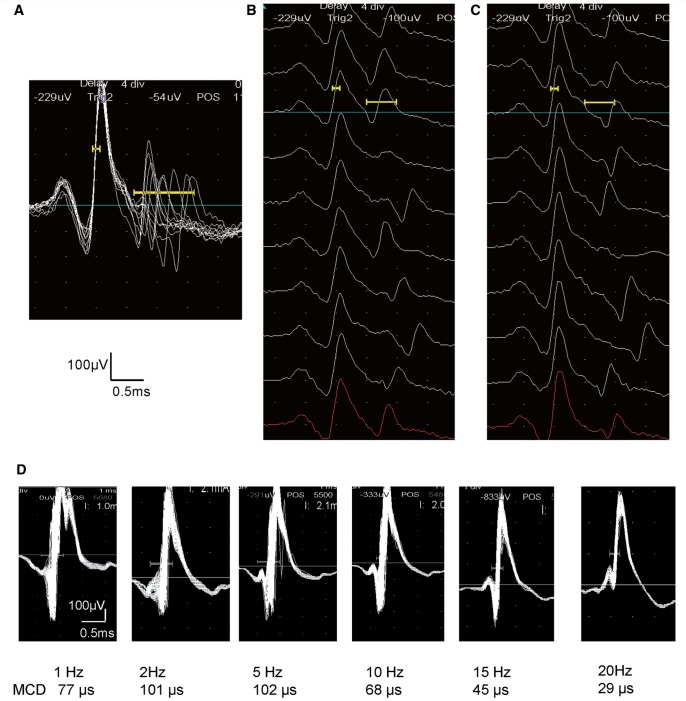
**Single-fibre EMG studies in EA2.** (**A–C**) Increased jitter with blocking in Subject 5 (Family 2). (**A**) Superimposed traces of single fibre EMG during recordings during voluntary contraction demonstrating increased jitter, reflecting instability of the neuromuscular junction. (**B** and **C**) Same recording displayed in raster mode showing increased jitter and blocking. (**D**) Stimulation single-fibre EMG in EA2. Stimulation rates are 1, 2, 5, 10, 15 and 20 Hz. Maximal jitter is seen at 2–5 Hz stimulation, decreasing at higher rates, similar to the findings in Lambert-Eaton syndrome.

Nerve conduction studies and EMG were performed in six of eight subjects. No significant abnormalities were detected on concentric needle EMG, nerve conduction or on repetitive stimulation of motor nerves at various rates. However, increased jitter was demonstrated on single fibre EMG (Fig. 1A) in Subjects 3 and 5 from Family 2, both of whom reported weakness during attacks. In addition, Subject 5 (in Family 2) had increased jitter with blocking on stimulated single fibre EMG, which was maximal at low stimulation rates (2–5 Hz; Fig. 1B). These findings demonstrate instability of neuromuscular transmission in these subjects. 

### Genetic results

New mutations were found in Families A and B (Table 1). In Family A, the mutation in exon 24: c.3992+1G>A p.? affects a consensus splice donor site. The consequence of this change is not predictable but is likely to cause a skip of exon 24, which would introduce a premature stop after the inclusion of three aberrant amino acids, which in turn predicts subsequent nonsense mediated decay of the aberrant message and may prevent translation of the mRNA.

The mutation identified in Family 2 is c.5137-10_2delAATTCCACA p.?. The consequence of this change is not predictable but is likely to cause a skip of exon 34, which would introduce a premature stop after the inclusion of 20 aberrant amino acids.

Subject 6 was known to carry G to A substitution at donor splice site of exon 21 (c.3695+1G>A p.?) (Eunson *et al.*, 2005). The consequence of this change again is not predictable, but is likely to cause a skip of exon 21, which would introduce a premature stop after the inclusion of nine aberrant amino acids.

Subject 7 carries a mutation that introduces a premature stop codon at the beginning of the C-terminus, c.5455C>T p.Arg1819*, which was not identified in his parents. This mutation has been previously reported in two unrelated patients in association with EA2, absence seizures and cognitive impairment (Jouvenceau *et al.*, 2001; Strupp *et al.*, 2004) albeit as Arg1820* due to a different reference sequence being used. Subject 8 carries the previously reported c.835C>T p.(Arg279Cys) mutation.

### Nerve excitability studies

The mean data for subjects with EA2 and normal controls are compared in Table 2. Prominent differences were found in absolute electrical thresholds, threshold electrotonus and the recovery cycle. 

**Table 2:** Comparison between nerve excitability measurements in EA2 and in normal controls

	Control	EA2	*t*(*df*)^a^	*P* *^b^*
	(*n* = 30)	(*n* = 8**)**		
**Stimulus-response and strength-duration properties**				
Stimulus (mA) for 50% maximal CMAP^c^	4.29 × /÷ 1.04	7.95 ×/÷ 1.19	3.49(7.59)	0.0090**
Peak CMAP (mV)^c^	8.66 ×/÷ 1.04	8.57 ×/÷ 1.05	0.14(22.8)	0.86
Strength-duration time constant (ms)	0.48 ± 0.018	0.45 ± 0.031	0.98(12.3)	0.35
Rheobase (mA)^c^	2.80 ×/÷ 1.04	5.33 ×/÷ 1.19	3.64(7.7)	0.0070**
**Threshold electrotonus**				
TEd^40^(peak) (%)	68.17 ± 0.70	72.28 ± 0.90	3.61(16.38)	0.0023**
TEd^40^(90 − 100 ms) (%)	43.96 ± 0.66	47.15 ± 1.16	2.39(12.01)	0.033*
TEd^20^(peak) (%)	38.19 ± 0.52	42.28 ± 0.82	4.21(13.41)	0.0010**
TEh^20^(90−100 ms) (%)	−47.1 ± 1.0	−56.7 ± 2.7	3.36(7.8)	0.010*
TEh^40^(20−40 ms) (%)	−91.11 ± 1.25	−100.2 ± 1.67	4.38(15.82)	0.00053***
TEh^40^(90−100 ms) (%)	−116.7 ± 2.77	−138.6 ± 3.19	5.18(18.99)	0.000068****
**Current-threshold properties**				
Resting I/V slope	0.607 ± 0.014	0.54 ± 0.031	1.96(10.18)	0.075
Minimum I/V slope	0.246 ± 0.008	0.224 ± 0.011	1.6(15.21)	0.13
Hyperpolarizing I/V slope	0.341 ± 0.010	0.377 ± 0.024	1.35(9.77)	0.21
**Recovery cycle**				
RRP (ms)^c^	2.95 ×/÷ 1.02	2.58 ×/÷ 1.02	4.64(20.79)	0.00017***
Superexcitability (%)	−23.05 ± 0.93	−30.39 ± 1.91	3.45(10.52)	0.0057**
Subexcitability (%)	14.4 ± 0.65	15.07 ± 0.76	0.66(18.76)	0.52
**Subjects/recording**				
Age (years)	39.1 ± 2.4 (30)	40.6 ± 4.5 (8)	0.30 (11.23)	0.76
Sex (male = 1, female = 2)	1.47 ± 0.09 (30)	1.5 ± 0.19 (8)	0.16(10.62)	0.85
Temperature	33.25 ± 0.17 (30)	33.86 ± 0.26 (8)	1.95(13.41)	0.070

For definition of excitability measurements see Glossary.

Values are mean ± SEM, except for four measurements^c^.

^a^ *t* and degrees of freedom for Welch’s unequal variance *t*-test.

^b^ *P* indicates probability for two-tailed test *P < 0.05, **P < 0.01, ***P < 0.001, ****P < 0.0001.

^c^Values were normalized by log conversion and given as geometric mean ×/÷ standard error expressed as a factor.

#### Stimulus–response and strength–duration relationship

Subjects with EA2 exhibited an increased threshold. The stimulus required to produce a CMAP 50% of the maximal response was ∼85% greater in patients than controls [listed in Table 2 as ‘Stimulus (mA) for 50% maximal CMAP’; *P* = 0.009].

**Figure 2:**
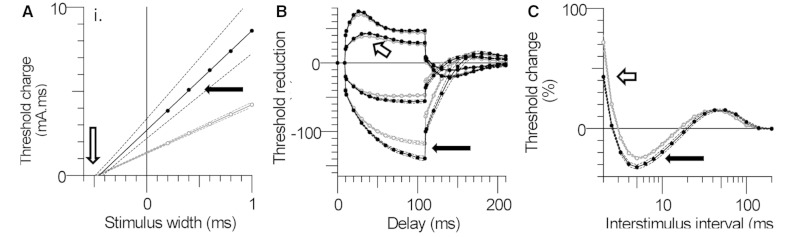
**Axonal excitability measurements in Subjects with EA2 compared to controls.** (**A**) Strength–duration time constant is given by the negative intercept of the regression line on the *x*-axis (open arrow). Rheobase is given by the slope of the regression line (filled arrow). (**B**) Threshold electrotonus in patients with EA2 and control subjects. Each point is mean ± SEM. Filled circles = EA2 (*n* = 8). Open arrow indicates increase in TEd in patients with EA2. Black arrow indicates increase in TEh in patients with EA2. (**C**) The recovery cycle in EA2. The patients’ recordings are characterized by a 12% shorter relative refractory period (open arrow) and a 24% increase in superexcitability (closed arrow).

**Figure 3:**
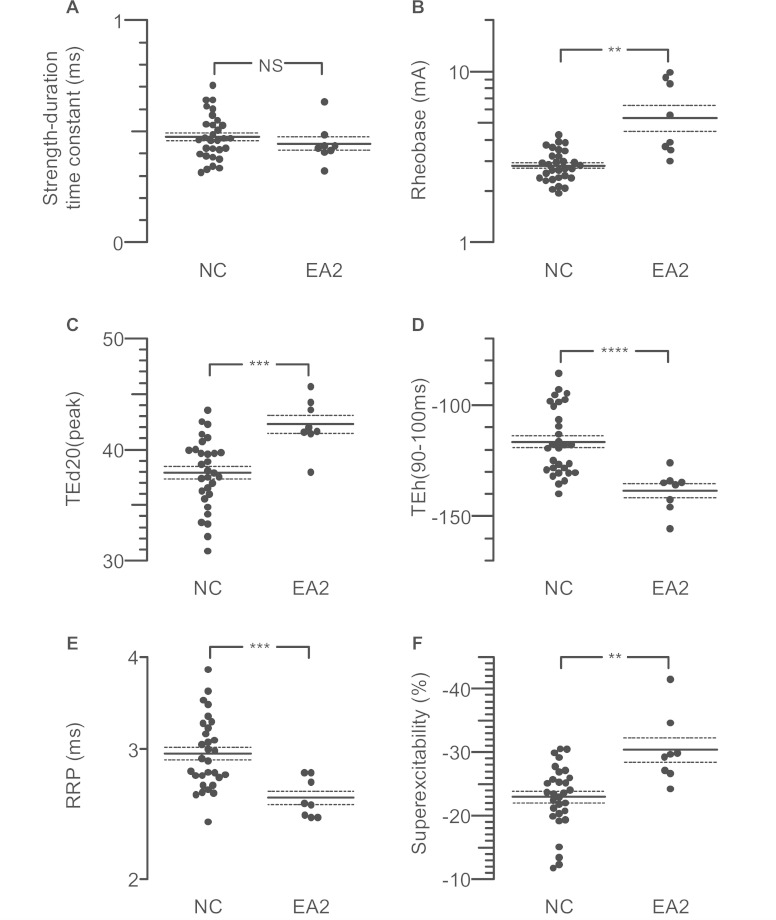
**Dot plots illustrating individual data points in EA2 subjects compared to controls.** (**A**) Strength duration time constant. (**B**) Rheobase. (**C**) Peak threshold decrease with 20% depolarizing current. (**D**) Threshold electrotonus with 40% hyperpolarizing current. (**E**) Relative refractory period (RRP). (**F**) Peak superexcitability in recovery cycle. NC = normal controls; NS = not significant. Asterisks indicate level of significance by Welch unequal variance *t*-test, as in Table 2. Note logarithmic axes in **B** and **E** to help normalize distributions. Horizontal lines indicate means (solid) and means ± standard error (dotted).

The strength–duration data are plotted in Fig. 2A as a charge–duration plot (i.e. mA.ms versus ms). The strength–duration time constant (τ_sd_) is given by the negative intercept of the regression line on the *x*-axis, and the rheobase is given by the slope of the regression line. The τ_sd_ was not significantly different (Figs 2A and 3A), but mean rheobase (i.e. threshold current for very long stimulus pulses) was nearly twice as high in the affected individuals (Table 2), and in four of eight cases was outside the 95% confidence limits for the normal controls (Fig. 3B). This is consistent with the increased threshold for activation, noted in the previous paragraph. 

#### Threshold electrotonus

Averaged threshold electrotonus curves for the eight patients are compared to the mean for the 30 controls (Fig. 2B). These graphs depict the change in threshold during and after 100 ms subthreshold conditioning currents of ± 40% and ±20% of the unconditioned threshold current. There was a greater increase in excitability in response to depolarizing currents in the patients and a greater decrease in excitability during hyperpolarization, producing a ‘fanning out’ appearance (Nodera and Kaji, 2006). These differences are statistically significant, especially for the hyperpolarizing threshold electrotonus (Fig. 3B and D). This is illustrated for TEd^20^(peak) (i.e. the greatest reduction in threshold during the 20% depolarizing current) in Fig. 3C and for TEh^40^(90–100 ms) (i.e. the increase in threshold at the end of the hyperpolarizing current, which is 40% of threshold) in Fig. 3D.

#### current–threshold relationship

The current–threshold relationship (Supplementary Fig. 1A) measures the changes in threshold at the end of 200 ms currents, varied in strength from 50% of threshold to −100% of threshold. Because threshold is closely related to membrane potential, this plot is a threshold analogue of a current–voltage curve (Kiernan *et al.*, 2000). Similarly, the slope of this curve is a threshold analogue of input conductance, designated ‘IV slope’ in Fig. 1B. There were only modest differences between the subjects with EA2 and controls (Table 2).

#### Recovery cycle

The excitability changes following supramaximal stimulation are compared for the patients with EA2 and control subjects in Fig. 2C. The patient recordings were characterized by a 13% shorter relative refractory period (white arrow) and a 30% greater superexcitability (black arrow). These differences are statistically significant (Fig 3E, F and Table 2), but there was no significant difference in late subexcitability.

### Modelling

**Figure 4:**
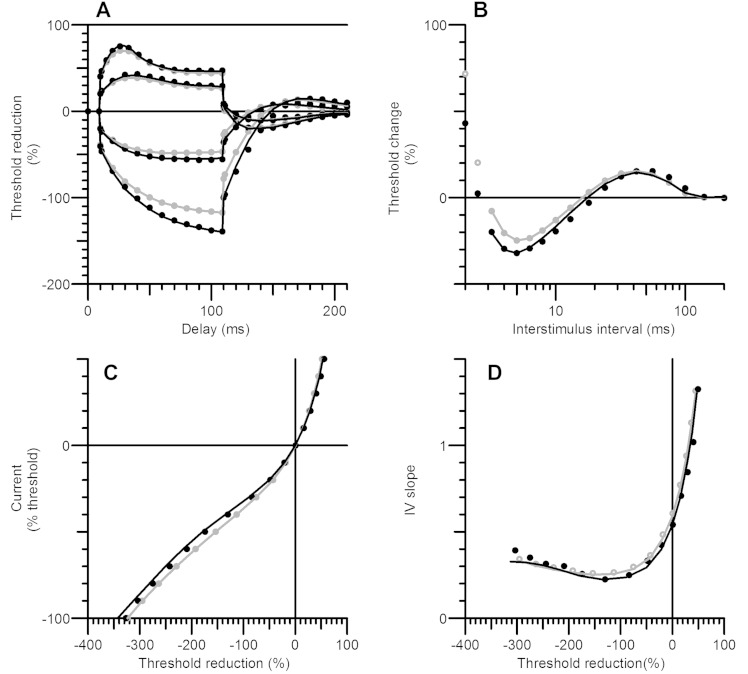
**Difference between control and EA2 nerve excitability modelled as an increase in Barrett-Barrett conductance.** The control results are in grey and the patient results in black, with the recorded data shown in circles and the output of the model by continuous lines. For the patient data the modelled change was a 13.7% increase in the Barrett-Barrett conductance. (**A**) Threshold electrotonus. (**B**) Recovery cycle. (**C**) Current/threshold reduction curve. (**D**) I/V slope.

After optimizing its parameters (see ‘Materials and methods’ section), the model fitted the control data with a residual discrepancy of 0.063. The discrepancy between this control model and the mean EA2 axonal excitability data was 1.15. To explore whether, for example, a change in fast potassium channels could account for this discrepancy, we determined the best reduction in discrepancy that could be achieved by changing the fast potassium conductance at nodes, internodes, or by making the same relative change in both. The discrepancy reductions for these changes are listed in Table 3 and were only 23.0, 22.3 and 23.9%, respectively, so they could not provide a convincing explanation for the altered excitability. Table 3 also lists the best discrepancy reductions achieved by changing the 13 other conductances, and also by changing the three capacitances in the model, the resting membrane potential, and also the half-activation voltage of the HCN channels. This membrane property is sensitive to intracellular cyclic nucleotides and other metabolic factors and most likely accounts for much of the variability in nerve excitability properties between normal subjects, and the changes in inward rectification reported in some pathologies (Jankelowitz *et al.*, 2007; Lin *et al.*, 2008;Howells *et al.*, 2012). Interestingly, the most appropriate changes in the model, resulting in a 73% reduction in discrepancy were made not by changes in any ion channel, but by increasing the Barrett-Barrett conductance (the conductance of the multiple pathways between the internodal axolemma and the exterior of the nerve fibre; see Barrett and Barrett, 1982). Figure 4 illustrates the excitability curves generated by the control model and by the model for patient data. The control results are in grey and the patient results in black, with the recorded data shown in circles and the output of the model by continuous lines. For the patient data the modelled change was a 13.7% increase in the Barrett-Barrett conductance. The differences in excitability waveforms resemble those seen in the recordings from controls and patients in Fig. 2. For completeness, we also computed the best discrepancy reductions achievable by altering the remaining 14 channel gating parameters in the model (which are not listed in Table 3, as there was no reason to suspect they might be involved in EA2), but none of them reduced the discrepancy by more than 31%. 

**Table 3:** Modelling possible explanations of altered nerve excitability properties in EA2.

Model parameter	Control value	Best fit value	Units	Discrepancy	Discrepancy reduction (%)
Barrett-Barrett conductance	36.5	41.5	nS	0.311	73.0
Resting potential (altered by applied current)	−82.3	−83.4	mV	0.483	58.1
Internodal leak conductance	3.6	1.83	nS	0.503	56.3
Nodal and internodal leak conductances (rel)	1.0	0.61	-	0.504	56.3
Nodal leak conductance	1.08	0.17	nS	0.674	41.5
HCN channel half-activation voltage	−103.1	−109.9	mV	0.799	30.2
Slow potassium conductances (rel)	1.0	0.835	-	0.832	27.9
Nodal slow potassium conductance	50.8	42.3	nS	0.834	27.7
Fast potassium conductances (rel)	1.0	0.83	-	0.878	23.9
Nodal fast potassium conductance	19.9	15.7	nS	0.887	23.0
Internodal fast potassium conductance	100	62	nS	0.895	22.3
Persistent sodium permeability (% transient)	1.02	1.12	%	0.956	17.0
HCN channel conductance	5.15	3.5	nS	0.960	16.8
Transient sodium permeability	3.75	4.0	cm^3^s^−1^.10^−9^	0.998	13.4
Internodal slow potassium conductance	0.33	0.40	nS	1.12	2.2
Capacitance internodal axon	0.273	0.257	nF	1.13	1.6
Capacitance node	0.50	0.53	pF	1.15	0.6
Capacitance myelin	1.55	1.55	pF	1.15	0.0

List of best fits to the EA2 patient nerve excitability data made by changing a single parameter in the model first fitted to the normal control data. For example, increasing the Barrett-Barrett conductance from 36.5 to 41.5 nS reduces the discrepancy between the model and the EA2 data from 1.15 to 0.311, a reduction of 73%. The parameters designated ‘(rel)’ are factors applied to both nodal and internodal conductances of the same type.

## Discussion

The present study has revealed disturbed excitability of motor axons in eight subjects with genetically confirmed EA2, with changes that can be modelled by an increase in shunt conductance between the internodal axolemma and exterior.

All subjects reported the onset of characteristic attacks of cerebellar dysfunction lasting hours to days from childhood. Two subjects from one kindred described myasthenic-type weakness during attacks. All subjects experienced a clinical response to acetazolamide though this was incomplete in two. It could be anticipated that acetazolamide would have affected measures of axonal excitability, but this is unlikely to be significant at clinical dosages. It is therefore important that the studies in the two patients not taking acetazolamide did not differ significantly from those taking the medication at the time of the study. Three of eight subjects had subtle fixed cerebellar signs between attacks. Cerebellar ataxia is consistent with dysfunction of central synapses; however, Ca_v_2.1 is also expressed in peripheral motor axons, albeit confined to the neuromuscular junction (Vogel and Schwarz, 1995). Nevertheless, the measurements of axonal excitability at the wrist detected significant differences between the EA2 cohort and normal control subjects. The possible reasons for these altered excitability properties are discussed below.

### Abnormal neuromuscular transmission in episodic ataxia type 2

Ca_v_2.1 is highly expressed at the neuromuscular junction where it is closely associated with neurotransmitter exocytic machinery (Protti *et al.*, 1996; Plant *et al.*, 1998). As a result of an action potential, Ca_v_2.1 activation facilitates calcium-dependant acetylcholine release. In the autoimmune disease Lambert-Eaton myasthenic syndrome (LEMS), antibodies directed against Ca_v_2.1 impair presynaptic acetylcholine release producing muscle weakness. In these patients, the jitter and blocking is maximal at stimulation rates of ≤5 Hz and reduces at higher rates (Trontelj and Stålberg, 1991; Mills, 2005). The stimulated single fibre EMG findings in Subject 5 (in Family 2) closely parallel the findings in LEMS and is consistent with impaired Ca_v_2.1 function. Repetitive activation at high frequency (e.g. 20–50 Hz) restores neurotransmission (Mills, 2005). It is not surprising therefore that similar clinical and neurophysiological findings have been reported in patients with EA2. In the recovery cycle, it is conceivable that the conditioning discharge would affect transmission at the neuromuscular junction. In patients with LEMS, this could change the test CMAP at the intervals used in the recovery cycle (>5 Hz), producing equivalent changes in threshold. However, the two patients who had abnormal stimulated single fibre EMG had no abnormalities on repetitive stimulation and their recovery cycle data were not significantly different from those of the other six patients.

#### Axonal excitability in episodic ataxia type 2

This paper reports alterations in the excitability properties of motor axons in the nerve trunk in patients with EA2. Acetazolamide was not discontinued prior to the study, but as noted above, the same excitability changes were seen in the two patients in whom the medication had been discontinued for medical reasons. The features seen in patients with EA2 include an increase in threshold, a shortened relative refractory period and greater threshold changes to the changes in membrane potential seen in threshold electrotonus. These abnormalities are qualitatively similar to those in EA1 (Tomlinson *et al.*, 2010). However, some measures behaved differently, and this was not just qualitative. For example, late subexcitability was normal in EA2 (Table 2) whereas it was increased in the 20 patients with EA1, so that the difference between the two types of episodic ataxia was highly significant (EA2: 14.4 ± 0.65%; EA1: 24.1 ± 1.7%; mean ± SE, *P* < 0.0001, Welch test). There was greater accommodation to 40% depolarizing currents in EA1 (EA2: 15.07 ± 0.7%; EA1: 23.41 ± 1.59% *P* < 0.00001). Also, although superexcitability was increased in both groups, it was increased much more in EA1 (EA2: −30.39 ± 1.9%; EA1: −44.3 ± 1.8%, *P* < 0.0001). Low serum potassium can produce some of the features seen in EA2 (Boërio *et al.*, 2014). These include increased threshold change to hyperpolarization in threshold electrotonus and increased superexcitability. However, low serum potassium would have also increased the threshold change to depolarization and increased late subexcitability. These changes were not seen in the EA2 cohort.

#### Underlying mechanisms

Whereas the nerve excitability changes in the EA1 subjects can be attributed directly to the loss of K_v_1.1 fast potassium channels (Tomlinson *et al.*, 2010), the same mechanism cannot be responsible for the superficially similar but less severe changes in EA2. A quantitative model of motor nerve excitability, previously used to account for the excitability changes induced by puffer fish poisoning (Kiernan *et al.*, 2005*a*), showed that the single parameter change that best explained the differences between patients and controls was a 14% increase in the conductance path across and around the myelin sheath, also known as the Barrett-Barrett conductance (Barrett and Barrett, 1982) (Table 3 and Fig. 4). The modelling suggests that the altered axonal excitability arises from quite different mechanisms in EA1 and EA2, and in EA2 is more likely due to changes in Schwann cell ensheathment than in axonal ion channels. According to this interpretation, the superficial similarity in excitability changes in threshold electrotonus and in the recovery cycle arises because in each case, both applied currents and the current generated during the nerve impulse cause a greater change in the membrane potential of the internodal axon: in EA1 this is because the internodal axolemma has fewer K_v_1.1 channels to resist potential change, whereas in EA2 the access resistance restricting current flow to the internodes is lower. Comparison between Fig. 4 and Fig. 2 show that there is a good correspondence between the other excitability abnormalities recorded in EA2 and the predicted effects of increasing the Barrett-Barrett conductance, supporting the high discrepancy reduction for this parameter [which at 73% was almost as great as the 75% discrepancy reduction reported by Kiernan *et al.* (2005*a*) when recordings from patients with puffer fish poisoning were modelled by a reduction in sodium channel permeability]. While this level of ‘goodness of fit’ obtained by changing the Barrett-Barrett conductance provides a strong pointer to the nature of the abnormality, it cannot prove that this pathway is involved, and to find a plausible mechanism relating this interpretation to the calcium channel defect we must look elsewhere. Such a mechanism is suggested by the report of Alix *et al.* (2008), who observed transient expression of α1A subunit of P/Q calcium channels along the axolemma of optic nerve axons during early neonatal development in mice. Mutation in the *CACNA1A* gene caused malformation of the nodes of Ranvier, with disordered localization of nodal proteins including voltage-gated sodium channels, β IV spectrin and contactin-associated protein-1 (CASPR1, encoded by *CNTNAP1*). If the P/Q calcium channels present in peripheral nerve axons during development have a similar role, it is likely that the changes in excitability in peripheral nerve axons described here reflect the sustained impact of nodal malformation in the early neonatal period. The mathematical modelling is consistent with this hypothesis. Interestingly, studies from infancy to adulthood by Farrar *et al.* (2013) have demonstrated changes in excitability that are generally consistent with those described in EA2, and were attributed to maturational changes at the axo–glial junction.

Glossary

**Accommodation half-time:** Half-time of accommodative response to a 100 ms subthreshold depolarizing conditioning stimulus

**HCN:** Hyperpolarization activated, cyclic-nucleotide gated channels

**TEd:** Change in threshold in response to a subthreshold depolarising conditioning stimulus

**TEh:** Change in threshold in response to a subthreshold hyperpolarising conditioning stimulus

**I/V:** Current/voltage; the current/threshold relationship is analogous to the traditional current/voltage relationship

**I/V slope:** Slope of the current–threshold relationship

***I_h_*:** Hyperpolarization-activated current (mediated by HCN channels)

In conclusion, the excitability properties of peripheral motor axons in EA2 differ significantly from those in normal controls, and also from those in EA1. The pattern of excitability changes in EA2 may result from nodal malformation in the neonatal period. As was the case with our previous study of patients recovered from benign familial neonatal epilepsy (Tomlinson *et al.*, 2012), this study demonstrates the power of excitability studies to detect membrane abnormalities that are too subtle to produce symptoms or to be detected by routine neurophysiological studies. In addition, this study raises a further mechanism through which peripheral nerve axons can be altered in disorders that are primarily central (Kiernan *et al.*, 2005*b*; Jankelowitz *et al.*, 2007; Ng *et al.*, 2008;Boland *et al.*, 2009; Tomlinson *et al.*, 2010, 2012) even when the relevant channel is not normally expressed at the site of stimulation. Indirect consequences of inherited channelopathies should be taken into account when considering the circuit consequences of the ion channel defect. However, in their recent study, Zhang and David (2016) used Ca_2+_ imaging to provide evidence for T-type (Ca_v_3.x) Ca_2+_ channels at the node of Ranvier and L-type (Ca_v_1.x) channels on the paranodal or juxtaparanodal membrane of myelinated motor axons of the mouse. When axons were stimulated at 50 Hz for 10 s, there was a small increase in axoplasmic [Ca_2+_] at the node, and this was greater when there was paranodal demyelination. They found no evidence for the involvement of Ca_v_2.1 channels, so our conclusions are not directly affected, but it can no longer be maintained with confidence that Ca_2+_ channels are not present at the node of Ranvier of human motor axons.

## Supplementary Material

Supplementary Data

Supplementary Fig. 1A

## Abbreviations

CMAP: compound muscle action potential
EA1/2: episodic ataxia type 1/2

## References

[awv380-B1] Alix JJ Dolphin AC Fern R Vesicular apparatus, including functional calcium channels, are present in developing rodent optic nerve axons and are required for normal node of Ranvier formation. J Physiol 2008; 586: 4069–8409. 18599536 10.1113/jphysiol.2008.155077 PMC2652192

[awv380-B6] Barrett EF Barrett JN Intracellular recording from vertebrate myelinated axons: mechanism of the depolarising afterpotential. J Physiol 1982; 323: 117–44. 6980272 10.1113/jphysiol.1982.sp014064 PMC1250348

[awv380-B7] Battistini S Stenirri S Piatti M Gelf Ci Righetti P Rocchi R A new CACNA1A gene mutation in acetazolamide-responsive familial hemiplegic migraine and ataxia. Neurology 1999; 53: 38–43. 10408534 10.1212/wnl.53.1.38

[awv380-B8] Boërio D Bostock H Spescha R Z'Graggen WJ Potassium and the excitability properties of normal human motor axons in vivo. PLoS One 2014; 9: e98262. 24893161 10.1371/journal.pone.0098262 PMC4043986

[awv380-B9] Boland RA Bostock H Kiernan MC Plasticity of lower limb motor axons after cervical cord injury. Clin Neurophysiol 2009; 120: 204–9. 19036634 10.1016/j.clinph.2008.10.009

[awv380-B10] Bostock H Cikurel K Burke D Threshold tracking techniques in the study of human peripheral nerve. Muscle Nerve 1998; 21: 137–58. 9466589 10.1002/(sici)1097-4598(199802)21:2<137::aid-mus1>3.0.co;2-c

[awv380-B101] Bostock H Rothwell JC Latent addition in motor and sensory fibres of human peripheral nerve. J Physiol 1997; 498: 277–294. 9023784 10.1113/jphysiol.1997.sp021857 PMC1159250

[awv380-B102] Bostock H Sharief MK Reid G Murray NM Axonal ion channel dysfunction in amyotrophic lateral sclerosis. Brain 1995; 118: 217–25. 7534598 10.1093/brain/118.1.217

[awv380-B11] Burke D Howells J Trevillion L McNulty PA Jankelowitz SK Kiernan MC Threshold behaviour of human axons explored using subthreshold perturbations to membrane potential. J Physiol 2009; 587: 491–504. 19047204 10.1113/jphysiol.2008.163170 PMC2670058

[awv380-B12] Burke D Kiernan MC Bostock H Excitability of human axons. Clin Neurophysiol 2001; 112: 1575–85. 11514239 10.1016/s1388-2457(01)00595-8

[awv380-B13] Day NC Wood SJ Ince PG Volsen SG Smith W Differential localization of voltage dependent calcium channel alpha1 subunits at the human and rat neuromuscular junction. J Neurosci 1997; 17: 6226–6235. 9236233 10.1523/JNEUROSCI.17-16-06226.1997 PMC6568369

[awv380-B14] Eunson LH Graves TD Hanna MG New calcium channel mutations predict aberrant RNA splicing in episodic ataxia. Neurology 2005; 65: 308–10. 16043807 10.1212/01.wnl.0000169020.82223.dd

[awv380-B103] Farrar M Park SB Lin CS Kiernan MC Evolution of peripheral nerve function in humans: novel insights from motor nerve excitability. J Physiol 2013; 591: 273–86. 23006483 10.1113/jphysiol.2012.240820 PMC3630785

[awv380-B15] Gancher ST Nutt JG Autosomal dominant episodic ataxia: a heterogeneous syndrome. Mov Disord 1986; 4: 239–53. 10.1002/mds.870010404 3504247

[awv380-B16] Howells J Trevillion L Bostock H Burke D The voltage dependence of I*_h_* in human myelinated axons. J Physiol 2012; 500: 1625–40. 10.1113/jphysiol.2011.225573 PMC3413487 22310314

[awv380-B17] Imbrici P Eunson LH Graves TD Bhatia KP Wadia NH Kullmann DM Late-onset episodic ataxia type 2 due to an in-frame insertion in CACNA1A. Neurology 2005; 65: 944–6. 16186543 10.1212/01.wnl.0000176069.64200.28

[awv380-B18] Imbrici P Jaffe SL Eunson LH Davies NP Herd C Robertson R Dysfunction of the brain calcium channel Ca_v_2.1 in absence epilepsy and episodic ataxia. Brain 2004; 127: 2682–2692. 15483044 10.1093/brain/awh301

[awv380-B19] Jankelowitz SK Howells J Burke D Plasticity of inwardly rectifying conductances following a corticospinal lesion in human subjects. J Physiol 2007; 581: 927–40. 17363389 10.1113/jphysiol.2006.123661 PMC2170828

[awv380-B20] Jen J Wan J Graves M Yu H Mock AF Coulin CJ Loss-of-function EA2 mutations are associated with impaired neuromuscular transmission. Neurology 2001; 57: 1843–8. 11723274 10.1212/wnl.57.10.1843

[awv380-B21] Jen J Yue Q Nelson SF Yu H Litt M Nutt J A novel nonsense mutation in CACNA1A causes episodic ataxia and hemiplegia. Neurology 1999; 53: 34–7. 10408533 10.1212/wnl.53.1.34

[awv380-B22] Jen JC Graves TD Hess EJ Hanna MG Griggs RC Baloh RW; the CINCH investigators. Primary episodic ataxias: diagnosis, pathogenesis and treatment. Brain 2007; 130: 2484–93. 17575281 10.1093/brain/awm126

[awv380-B23] Jouvenceau A Eunson LH Spauschus A Ramesh V Zuberi SM Kullmann DM Human epilepsy associated with dysfunction of the brain P/Q-type calcium channel. Lancet 2001; 358: 801–7. 11564488 10.1016/S0140-6736(01)05971-2

[awv380-B25] Kiernan MC Burke D Andersen KV Bostock H Multiple measures of axonal excitability: a new approach in clinical testing. Muscle Nerve 2000; 23: 399–409. 10679717 10.1002/(sici)1097-4598(200003)23:3<399::aid-mus12>3.0.co;2-g

[awv380-B26] Kiernan MC Isibister GK Lin CS Burke D Bostock H Acute tetrodotoxin-induced neurotoxicity after ingestion of puffer fish. Ann Neurol2005a; 57: 339–48. 15732107 10.1002/ana.20395

[awv380-B104] Kiernan MC Krishnan AV Lin CS Burke D Berkovic SF Mutation in the Na+ channel subunit SCN1B produces paradoxical changes in peripheral nerve excitability. Brain2005b; 128: 1841–46. 15857929 10.1093/brain/awh520

[awv380-B27] Kors EE Melberg A Vanmolkot KRJ Kumlien E Haan J Rainink R Childhood epilepsy, familial hemiplegic migraine, cerebellar ataxia, and a new CACNA1A mutation. Neurology 2004; 63: 1136–7. 15452324 10.1212/01.wnl.0000138571.48593.fc

[awv380-B28] Maselli RA Wan J Dunne V Graves M Balow RW Wollmann RL Presynaptic failure of neuromuscular transmission and synaptic remodeling in EA2. Neurology 2003; 61: 1743–8. 14694040 10.1212/01.wnl.0000099748.41130.9a

[awv380-B29] Melzer N Classen J Reiners K Buttmann M Fluctuating neuromuscular transmission defects and inverse acetazolamide response in episodic ataxia type 2 associated with the novel CaV2.1 single amino acid substitution R2090Q. J Neurol Sci 2010; 296: 104–6. 20663518 10.1016/j.jns.2010.06.024

[awv380-B30] Mills KR Specialised electromyography and nerve conduction studies. J Neurol Neurosurg Psychiatry 2005 (Suppl 2): ii36–40. 15961867 10.1136/jnnp.2005.068981 PMC1765690

[awv380-B31] Ng K Howells J Pollard JD Burke D Up-regulation of slow K(+) channels in peripheral motor axons: a transcriptional channelopathy in multiple sclerosis. Brain 2008; 131: 3062–371. 18697908 10.1093/brain/awn180

[awv380-B32] Nodera H Kaji R Nerve excitability testing and its clinical application to neuromuscular diseases. Clin Neurophysiol 2006; 117: 1902–16. 16631406 10.1016/j.clinph.2006.01.018

[awv380-B33] Ophoff RA Terwindt GM Vergouwe MN van Eijk R Oefner PJ Hoffman SM Familial hemiplegic migraine and episodic ataxia type 2 are caused by mutations in the Ca^2+^ channel gene. CACNL1A4. Cell 1996; 87: 543–52. 8898206 10.1016/s0092-8674(00)81373-2

[awv380-B34] Ousley AH Froehner SC An anti-peptide antibody specific for the class A calcium channel alpha 1 subunit labels mammalian neuromuscular junction. Proc Natl Acad Sci USA; 1994; 91: 12263–7. 7991616 10.1073/pnas.91.25.12263 PMC45417

[awv380-B35] Plant TD Schirra C Katz E Uchitel OD Konnerth A Single-cell RT-PCR and functional characterization of Ca2+ channels in motoneurons of the rat facial nucleus. J Neurosci 1998; 18: 9573–84. 9822718 10.1523/JNEUROSCI.18-23-09573.1998 PMC6793322

[awv380-B36] Protti DA Reisin R Mackinley TA Uchitel OD Calcium channel blockers and transmitter release at the normal human neuromuscular junction. Neurology 1996; 46: 1391–6. 8628488 10.1212/wnl.46.5.1391

[awv380-B37] Rajakulendran S Graves TD Labrum RW Kotzadimitriou D Eunson L Davis MB Genetic and functional characterisation of the P/Q calcium channel in episodic ataxia with epilepsy. J Physiol 2010; 588: 1905–13. 20156848 10.1113/jphysiol.2009.186437 PMC2901979

[awv380-B38] Schwarz JR Reid G Bostock H Action potentials and membrane currents in the human node of Ranvier*.* Pflügers Arch 1995; 430: 283–92. 7675638 10.1007/BF00374660

[awv380-B39] Strupp M Kalla R Dichgans M Freilinger T Glasauer S Brandt T Treatment of episodic ataxia type 2 with the potassium channel blocker 4-aminopyridine. Neurology 2004; 62: 1623–5. 15136697 10.1212/01.wnl.0000125691.74109.53

[awv380-B41] Tomlinson SE Bostock H Grinton B Hanna MG Kullmann DM Kiernan MC Nerve excitability studies characterise loss of slow potassium channel activity *in vivo* in patients with Benign Familial Neonatal Epilepsy in remission. Brain 2012; 135: 3144–52. 23065794 10.1093/brain/aws241 PMC3470715

[awv380-B42] Tomlinson SE Hanna MG Kullmann DM Tan SV Burke D Clinical neurophysiology of the episodic ataxias: insights into ion channel dysfunction in vivo. Clin Neurophysiol 2009; 120: 1768–76. 19734086 10.1016/j.clinph.2009.07.003

[awv380-B43] Tomlinson SE Tan SV Kullmann DM Griggs RC Hanna MG Burke D Nerve excitability studies characterize Kv1.1 fast potassium channel dysfunction in patients with episodic ataxia type 1. Brain 2010; 133: 3530–40. 21106501 10.1093/brain/awq318 PMC2995887

[awv380-B44] Trontelj JV Stalberg E Single motor end-plates in myasthenia gravis and LEMS at different firing rates. Muscle Nerve 1991; 14: 226–32. 1645844 10.1002/mus.880140305

[awv380-B46] Vacher H Mohapatra DP Trimmer JS Localization and targeting of voltage-dependent ion channels in mammalian central neurons. Physiol Rev 2008; 88: 1407–47 18923186 10.1152/physrev.00002.2008 PMC2587220

[awv380-B47] Vogel W Schwarz JR Voltage-clamp studies in axons: macroscopic and single channel currents. In: Waxman S Kosics JD Stys PK, editors. The axon; structure, function and pathophysiology. New York, NY: Oxford University Press; 1995 p. 257–80.

[awv380-B49] Westenbroek RE Hoskins L Catterall WA Localization of Ca^2+^ channel subtypes on rat spinal motor neurons, interneurons, and nerve terminals. J Neurosci 1998; 18: 6319–30. 9698323 10.1523/JNEUROSCI.18-16-06319.1998 PMC6793183

[awv380-B105] Zhang Z David G Stimulation-induced Ca^2+^ influx at nodes of Ranvier in mouse peripheral motor axons. J Physiol; 2016; 594: 39–57. 26365250 10.1113/JP271207 PMC4704498

