# Supplementary material for: In vivo impact of presynaptic calcium channel dysfunction on motor axons in episodic ataxia type 2
Source: Brain. 2016 Jan 27;139(2):380–91. doi: 10.1093/brain/awv380 (PMC4795516; doi:10.1093/brain/awv380)
Supplement: Supplementary Data [file awv380_supplementary_data.zip › brain-2015-00673-File011.pdf]

Supplementary figure 1

A. The current–threshold relationship illustrates the changes in threshold 200ms into prolonged polarising currents of different strengths.

B. The slope of the current-threshold curve is a threshold analogue of input conductance, designated 'IV slope'. There are only modest differences which are not statistically significant between the EA2 patients and controls (Table 2).
