# Supplementary material for: In vivo impact of presynaptic calcium channel dysfunction on motor axons in episodic ataxia type 2
Source: Brain. 2016 Jan 27;139(2):380–91. doi: 10.1093/brain/awv380 (PMC4795516; doi:10.1093/brain/awv380)
Supplement: Supplementary Data [file awv380_supplementary_data.zip › brain-2015-00673-File002.docx]

**Glossary:**

**Accommodation half-time:** Half-time of accommodative response to a 100 ms subthreshold depolarizing conditioning stimulus

**HCN:** Hyperpolarization activated, cyclic-nucleotide gated channels

**TEd:** Change in threshold in response to a subthreshold depolarising conditioning stimulus

**TEh:** Change in threshold in response to a subthreshold hyperpolarising conditioning stimulus

**I/V:** Current/voltage; the current/threshold relationship is analogous to the traditional current/voltage relationship

**I/V** **slope:** Slope of the current–threshold relationship

***I*_h_:** Hyperpolarization-activated current (mediated by HCN channels)
