# Supplementary figures and images for: In vivo impact of presynaptic calcium channel dysfunction on motor axons in episodic ataxia type 2
Source: Brain. 2016 Jan 27;139(2):380–91. doi: 10.1093/brain/awv380 (PMC4795516; doi:10.1093/brain/awv380)

A

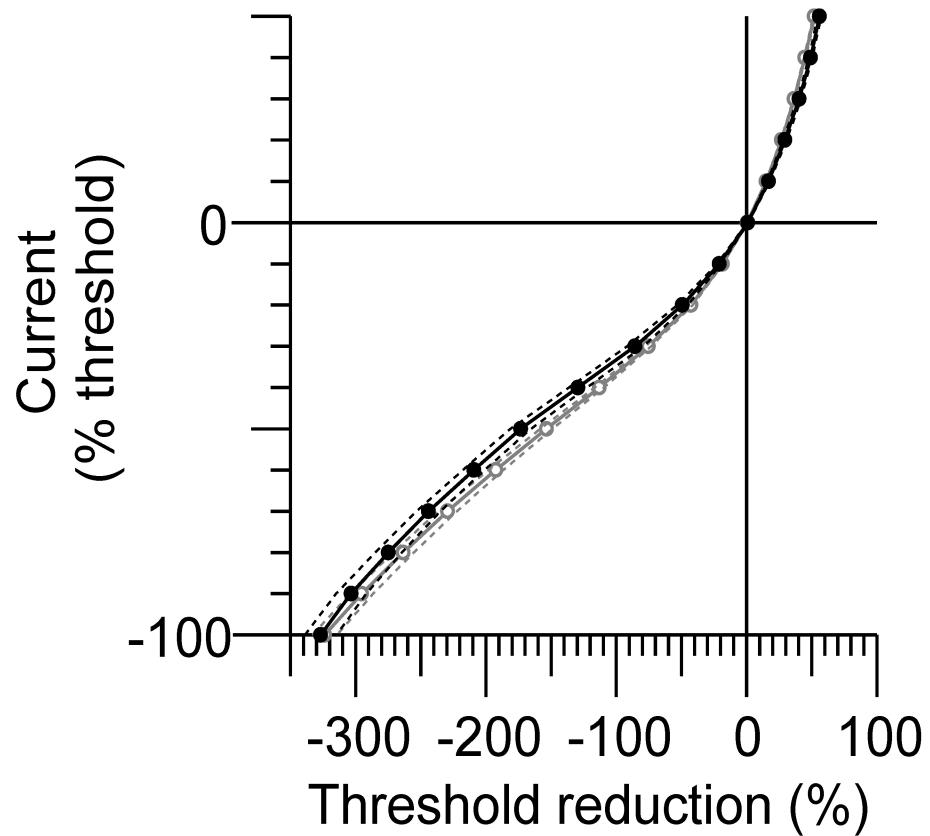

B

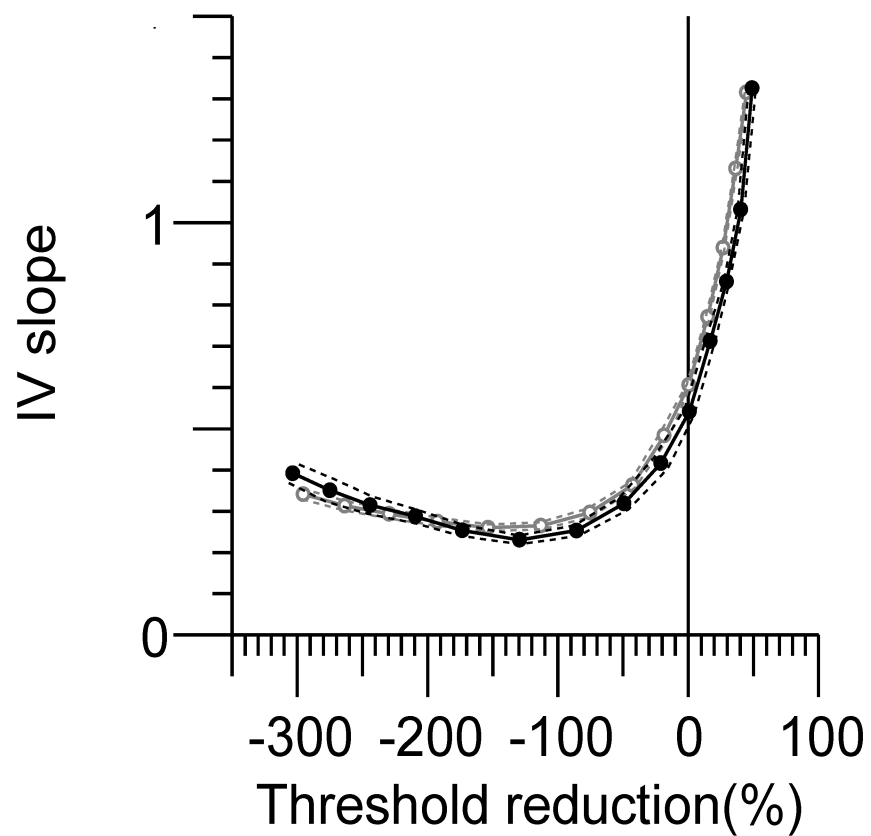

Supplement: Supplementary Data [file awv380_supplementary_data.zip › brain-2015-00673-File010.pdf]
